# Retrospective Staged Evaluation of an Optical Conveyor-Belt Monitoring System with On-Camera Processing: A Single-Site Mining Case Study

**DOI:** 10.3390/s26185925

**Published:** 2026-09-19

**Authors:** Daniel Luiz Souza, Geraldo Lucas Pugialli de Paiva, Filipe Bastos Vargas, Lucas Eugenio Ribeiro e Souza, Julio Barbosa, Luiz Henrique Jorge Machado

**Affiliations:** 1 Vale S.A., Complexo Itabira, Mina do Cauê, Estrada Serra do Esmeril, Itabira 35900-900, MG, Brazil; daniel.souza@vale.com; 2 LLK Engenharia, BH-TEC, Av. Prof. José Vieira de Mendonça, 770, Sala 501, Belo Horizonte 31270-901, MG, Brazil; geraldo.paiva@llk.com.br (G.L.P.d.P.); f.vargas@llk.com.br (F.B.V.); 3 Vale S.A., Alameda Oscar Niemeyer, 132, Suites 1501-3102, Nova Lima 34006-049, MG, Brazil; lucas.ribeiro.souza@vale.com (L.E.R.e.S.); julio.barbosa@vale.com (J.B.); 4 Department of Mechanical Engineering (DEMEC), Universidade Federal de Minas Gerais (UFMG), Av. Pres. Antônio Carlos, 6627, Belo Horizonte 31270-901, MG, Brazil; 5 Graduate Program in Technological Innovation (PPGIT), Universidade Federal de Minas Gerais (UFMG), Av. Pres. Antônio Carlos, 6627, Belo Horizonte 31270-901, MG, Brazil

**Keywords:** conveyor belt monitoring, optical sensing, condition monitoring, fault diagnosis, operational maturation, mining automation, industrial evaluation

## Abstract

Industrial sensor studies often report controlled defects or isolated field events without preserving the commissioning evidence that connects them. This retrospective single-site study evaluates an optical conveyor-belt monitoring system through technical assessment, industrial and belt-loop tests, control-system integration, six investigation groups, and one field-confirmed edge tear. The installation used upper and lower optical camera subsystems with embedded processing, proprietary internal confirmation logic, industrial communication, and connection to the plant programmable logic controller (PLC). Repeated passages of the same defects were treated as non-independent descriptive observations. Among the quantified configurations, scenario-specific descriptive PLC-output proportions ranged from 20.0% for an approximately 0.20-mm nearly closed central slit to 96.0% for a 6-mm V-shaped opening. Fourteen protection-output assertions were assigned to six investigations. Corrective observation preceded re-interlocking on 7 August. On 31 October, the edge-tear indication reached the configured protective-stop state and caused an automatic conveyor stop; inspection confirmed the edge tear, and maintenance repaired the belt. The case demonstrates why camera-interface recognition, confirmed output, plant action, and field confirmation must be reported separately. Internal optical calculations, confirmation parameters, software implementation, configuration details, and decision logic remain proprietary.

## 1. Introduction

Conveyor belts are critical production assets in mining and bulk-material handling because local damage can interrupt long transport chains and propagate under continuous loading. Belt damage includes edge deterioration, central cuts, longitudinal tears, splice anomalies, surface wear, and misalignment, with physical manifestations that depend on damage geometry, belt construction, operating load, and installation conditions [1,2]. Recent reviews show rapid growth in camera-based monitoring, laser-stripe analysis, machine learning, multisensor fusion, and data-driven diagnosis [3,4]. However, much of the published evidence remains algorithm-centric or laboratory-centered, and a high benchmark score does not by itself demonstrate reliable integration with industrial control, tolerance to contamination, configuration management, or defensible interpretation after sequential commissioning changes.

Optical methods are attractive because they provide non-contact access to visible geometry and can support localization and characterization. Laser-stripe and structured-light systems have been used to detect and quantify longitudinal rips [5,6,7], while image-based classifiers have been developed for surface damage and state monitoring [8,9]. Field deployment nevertheless introduces dust, carryback, variable reflectivity, vibration, occlusion, and maintenance constraints. Related work has explicitly shown the importance of dirt detection, sensor orientation, and industrial inspection architecture [10,11,12].

This study applies a source-traceable staged evidence structure to one optical monitoring installation with on-camera processing. The structure connects controlled physical defects, camera-interface recognition, proprietary internal confirmation logic, industrial-control integration, grouped operational investigations, controlled interlock transitions, and field-confirmed evidence. It is used here as an analytical structure for a single-site case study rather than presented as a universally validated framework. The contribution lies in system-level experimental and operational evaluation rather than in proposing a new general-purpose vision algorithm. The study addresses three questions:

1. How should repeated-pass controlled tests be interpreted without treating multiple passages of one defect as independent fault samples?
2. How can grouped protection-output assertions be reported when they are not equivalent to independent physical events and when consolidated and detailed source records use different timing labels?
3. What conclusions can be drawn from one field-confirmed, automatically interlocked damage event without claiming global accuracy, availability, or universal superiority?

The transferable contribution is the source-traceable separation of sensor recognition, internally confirmed output, control output, plant action, and physical confirmation across sequential configuration changes, not a universal performance estimate or a new vision algorithm. The main contributions are: (i) a source-traceable separation of camera-interface recognition, proprietary internal confirmation, programmable logic controller (PLC)-output assertion, plant-control action, and field confirmation; (ii) conservative repeated-pass results for five controlled defect/output scenarios without treating loops as independent fault samples; (iii) a bounded account of fourteen reported protection-output assertions assigned to six investigation groups, with source discrepancies disclosed and operational maturation reported through broad evidence categories; and (iv) a single field-confirmed edge-tear case in which the indication reached the configured protective-stop state and caused an automatic conveyor stop.

## 2. Related Work and Research Gap

Recent systematic and critical reviews cover conveyor-belt damage, sensing principles, and computer-vision methods [2,3,4]. Direct optical studies commonly report image-level or passage-level detection metrics under controlled conditions. Xu et al. used binocular line-laser stereo vision and three-dimensional point-cloud processing to identify and characterize longitudinal rips [5]. Qiao et al. developed a conditional-characteristics vision system for real-time tear detection [6]. Leite et al. combined a two-dimensional laser scanner with statistical processing [7]. More system-oriented studies have integrated machine vision with industrial sensors, PLC conditioning, communication networks, and remote monitoring [13]. Wang et al. reported a PLC-based laser-scanning architecture with industrial cameras, dust-control provisions, and automated monitoring [14]. Other studies emphasize learning-based surface-damage detection [8,9], sensor-based lifecycle diagnostics [15], or indirect indicators such as motor-current signatures [16].

Recent contributions to the first edition of this Special Issue illustrate complementary deployment concerns. Esmaili et al. proposed an embedded-compatible, cycle-synchronized monitoring framework for variable real-world machining without requiring controller or encoder access [17]. Shi et al. showed that industrial online-monitoring anomalies may reflect sensor faults, communication errors, or incipient equipment failures and therefore require interpretable localization before maintenance decisions are made [18].

These studies establish relevant sensing, integration, and inference methods. The less frequently reported element is a source-traceable sequence that retains initial deficiencies, controlled negative results, configuration changes, industrial-control integration, investigated protection-related occurrences, and bounded field confirmation within one deployment. The present work therefore treats commissioning and deployment evidence as an object of analysis. It separates: (i) visibility or geometric recognition in the sensor interface; (ii) internally confirmed output sent to the programmable logic controller (PLC); (iii) subsequent operator or plant-control action; and (iv) field confirmation. This separation prevents recognition, PLC-output assertion, plant action, and independent event detection from being treated as equivalent quantities. It is also consistent with the layered treatment of condition-monitoring programs and of data processing, communication, and presentation in ISO 17359 and ISO 13374-1 [19,20].

Relative to the representative studies summarized in Table 1, the novelty of the present work does not reside in any individual optical, communication, or PLC component. It resides in preserving and reconciling at the source level, within the same sequentially changing industrial deployment, negative controlled evidence, repeated-pass test evidence, industrial-control integration, grouped operational occurrences, controlled interlock transitions, and bounded physical field confirmation. This combined evidentiary sequence is the specific scientific contribution evaluated in this study.

Table 1 places the present contribution in the context of the closest optical studies. The comparison is intentionally limited to sensing architecture, validation setting, and reported output because performance values obtained from different damage geometries and sampling units are not directly interchangeable.

## 3. Materials and Methods

### 3.1. Optical Principle, Functional Measurands, and Validity States

The system uses non-contact optical sensing and embedded camera processing to evaluate accessible belt manifestations. The industrial installation comprises an upper subsystem (Face) with two laterally separated cameras at one longitudinal station and a lower subsystem (Contraface) with one camera. During commissioning, calibrated reference positions and optical conditions were established for the installed arrangement. After analysis relative to this calibrated condition, the Face subsystem provides indications associated with edge tear and misalignment, while the Contraface subsystem provides indications associated with accessible central manifestations. Both subsystems also evaluate surface texture and optical-data validity. The calculation method, optical geometry, illumination arrangement, acquisition parameters, calibration implementation, feature extraction, confirmation parameters, and state-transition logic remain proprietary.

**Figure 1:**
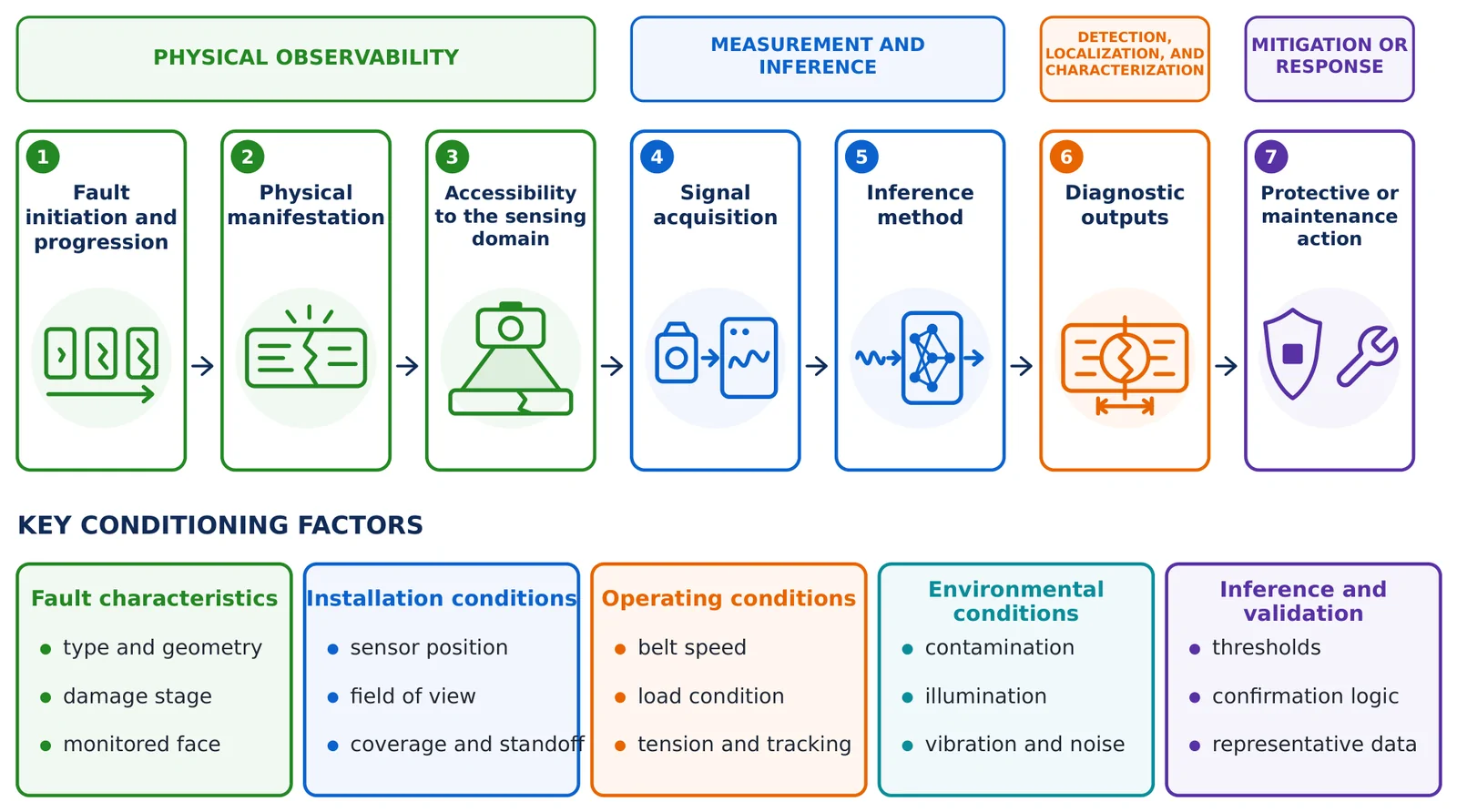
Conceptual source-to-action framework separating physical observability; measurement and inference; detection, localization, and characterization; and mitigation or response. The lower panel summarizes conditioning factors related to fault characteristics, installation conditions, operating conditions, environmental conditions, and inference and validation.

**Figure 2:**
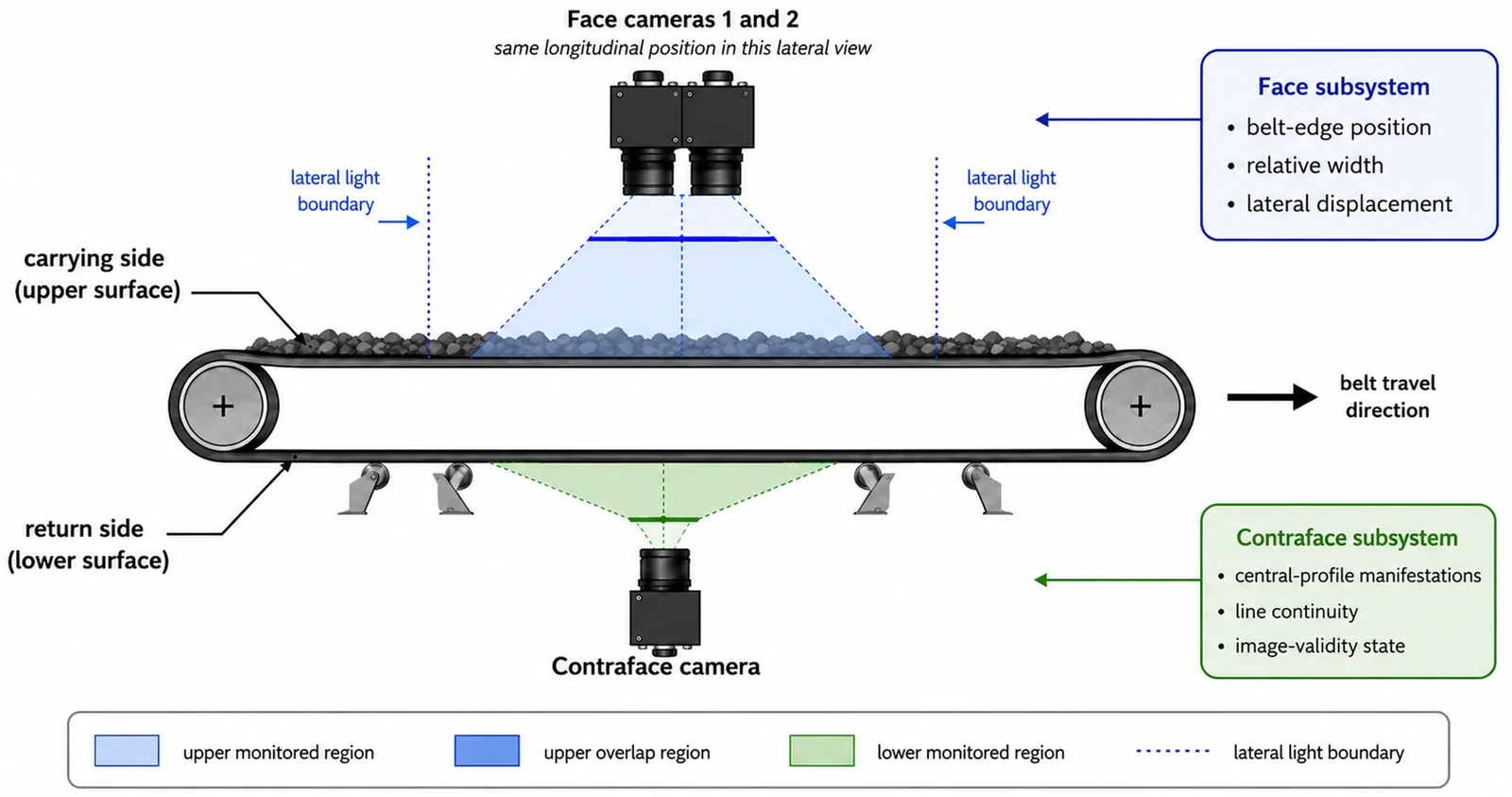
Publishable physical arrangement of the optical monitoring station in lateral view. The upper Face subsystem comprises two laterally separated cameras at one longitudinal station, and the lower Contraface subsystem observes the return side. The shaded regions are functional indications only and do not represent optical fields of view. Optical geometry, illumination arrangement, standoff, calibration, acquisition parameters, internal processing, and decision criteria are intentionally omitted.

**Figure 3:**
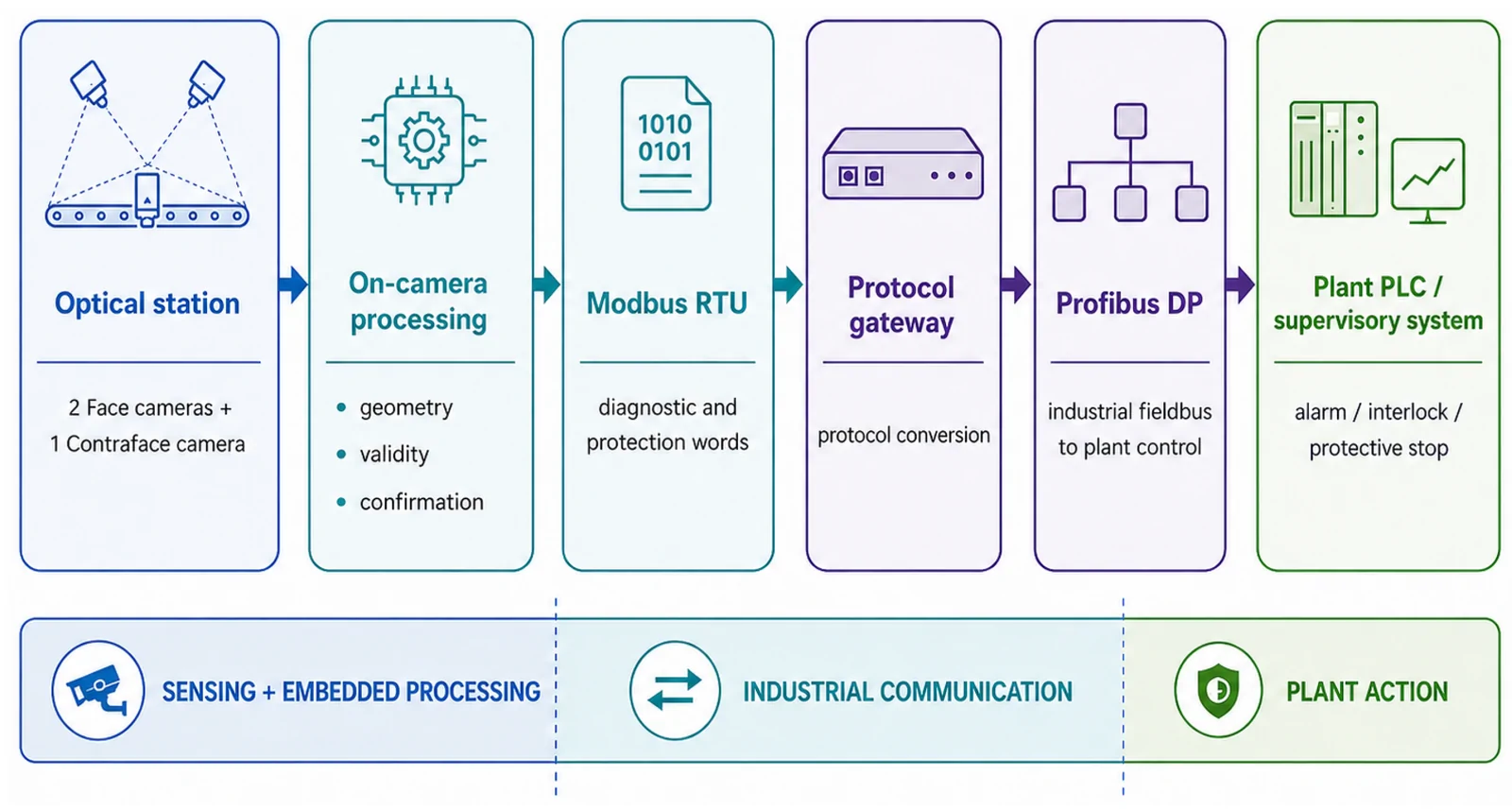
Functional architecture of the optical monitoring and industrial-control chain. The protocol gateway performs protocol conversion between Modbus RTU and Profibus DP before transfer to the plant PLC/supervisory environment. The gateway model, register maps, network identifiers, internal confirmation parameters, thresholds, calibration constants, and software implementation are intentionally omitted.

An observation is functionally usable when the optical characteristics required by the relevant subsystem pass internal validity checks. Missing or invalid optical information generates diagnostic states rather than a confirmed tear-related output. During commissioning, the defect-validation and internal confirmation logic were improved to increase robustness across the documented operating conditions. Implementation parameters, calculation details, tolerances, calibration, thresholds, corrective implementation, and state-transition criteria are not disclosed. The study therefore evaluates externally auditable input–output behavior, integration, and deployment evidence, but does not enable independent replication of the proprietary sensing and decision implementation. Figure 1 summarizes the conceptual source-to-action separation used in the study, Figure 2 depicts the publishable physical arrangement of the three-camera station, and Figure 3 presents the documented functional sensor-to-control communication chain. Table 2 summarizes the functional measurands and externally observable outputs of the optical subsystems.

### 3.2. Publishable System Specification and Replication Boundary

Table 3 consolidates the implementation details that can be supported by the retained records and published without exposing proprietary decision thresholds, calibration constants, source code, or industrial network identifiers. The retained evidence supports audit of the reported sensing and control chain and independent recalculation of the published input–output results. It does not support implementation-level reconstruction, independent physical replication of the optical subsystem, or component-level replication of the proprietary image-processing implementation.

### 3.3. Diagnostic and Protective Outputs

The integrated interface transmitted hardware, optical-validity, communication, alarm, protective, and alignment-related states to the plant PLC and supervisory environment through Modbus RTU, a protocol gateway, and Profibus DP. The integration tests verified transfer of the configured diagnostic and protective outputs. Register assignments, network identifiers, and alternative integration arrangements are retained confidentially. These tests demonstrate communication functionality; they do not constitute defect-detection performance tests.

In the retained installation, a configured protective-stop state was assigned to initiate the automatic conveyor-stop function. The internal nomenclature and intensity thresholds are not required for interpretation of the published evidence and are not disclosed.

### 3.4. Retrospective Evidence Design and Staged Evaluation

#### 3.4.1. Evidence Sources, Inclusion Boundaries, and Verification

This retrospective single-site study included records generated from March through 4 November 2024 for the same monitoring installation and its associated controlled tests. Eligible evidence comprised the initial technical assessment, the May controlled industrial test, the June belt-loop tests, the integration report, the final assisted-operation report and its embedded detailed investigation annexes, retained historian extracts, PLC states, field inspection, and maintenance confirmation. Draft presentations and performance claims without a traceable primary record were excluded from quantitative analysis. Table 4 summarizes the evidence stages. Internal report identifiers are retained in the confidential audit package but are anonymized here as Reports A–E.

The consolidated assisted-operation report was used for the total count of protection-output assertions and its six investigation-group labels. The corresponding detailed annexes were used confidentially to verify interlock context, source relationships, and broad operational-maturation categories. Some consolidated labels did not coincide with the timing in the detailed records. These differences are disclosed in the Supplementary Materials without reproducing implementation-specific mechanisms, software changes, or corrective algorithms, and no unsupported single event date was created. Values were first extracted into source-level tables and then checked in a second pass against the primary record. When records differed in detail or completeness, the narrower claim was retained. Vale S.A. and LLK Engenharia authorized the scientific use of the anonymized records, derived data, and images included in this study.

#### 3.4.2. Controlled-Test Configuration

The belt-loop test rig consisted of an approximately 18.4-m belt loop, a nominal belt width of 610 mm, and a belt speed of 1.9 m/s. The system monitored upper and lower regions and transmitted confirmed outputs to a PLC. Proprietary internal confirmation and defect-validation logic was active during the documented tests. Its internal parameters and implementation are not disclosed. The bench and industrial stages are treated as complementary evidence rather than dynamically equivalent configurations, and no direct scale-transfer relationship or controlled validation at the industrial belt speed is claimed.

The controlled configurations included: (i) a nominal 35-mm edge tear below the first output threshold; (ii) a nominal 61-mm edge tear; (iii) a nominal 90-mm edge tear evaluated at two output levels; (iv) a nearly closed central slit with an opening estimated at approximately 0.20 mm; and (v) a nominal 6-mm V-shaped central opening. Edge and central defects were approximately 1000 mm long. The central-slit opening was not measured directly because the caliper jaws could not be inserted. It was estimated using a rigid card that fitted tightly into the slit. Five caliper measurements of the card thickness yielded 0.20±0.02 mm. Because the slit opening itself was not directly measured, this dispersion is not interpreted as a formal uncertainty in the slit opening. The remaining dimensions were nominal values documented in the controlled-test procedure; individual dimensional uncertainties were not retained. The central slit was produced in a new, unloaded belt, so its opening was not enlarged by transported material or operational surface tension. The same physical defect repeatedly passed through the monitored region; therefore, the passage counts are not independent defect samples.

#### 3.4.3. Counting Procedure and Descriptive Proportions

Before the belt-loop tests, the system inputs were set to a nominal belt width of 610 mm and a belt speed of 1.9 m/s. The 14 June scenarios were planned for approximately 30 min, corresponding to the final documented count of 185 belt revolutions per quantified scenario. The 23 June V-opening scenario was operated for 8 min and 10 s, corresponding to 50 documented revolutions. A passage was therefore operationally represented by one completed belt-loop revolution of the same physical defect specimen through the fixed sensing station. No additional pass-level acceptance or exclusion criterion was preserved in the retained report.

For scenario *j*, $n_{P,j}$ denotes the documented number of belt passages. The recognition count $n_{R,j}$ was obtained from the camera-system interface counters documented during the test, while the confirmed-output count $n_{PLC,j}$ was obtained from dedicated PLC counters. The report preserved the final counter values and scenario totals but did not retain synchronized row-by-row passage timestamps, the counter-reset sequence, a pass-level operator log, ambient-light or contamination measurements, a cleaning record between scenarios, or a list of discarded passages. Consequently, the published denominators and numerators are the final documented integer counts; no undocumented exclusions, resets, or environmental corrections were introduced. The observed pass-level recognition proportion was

(1) $${\hat{p}}_{R,j}=\frac{n_{R,j}}{n_{P,j}},$$

and the pass-level PLC-output-assertion proportion was

(2) $${\hat{p}}_{PLC,j}=\frac{n_{PLC,j}}{n_{P,j}}.$$

when recognition was recorded separately, the descriptive PLC-output-to-recognition count ratio was $n_{PLC,j}/n_{R,j}$. The same physical specimen circulated repeatedly, so passages were non-independent repeated observations of the same physical specimen rather than independent experimental replicates. Inferential confidence intervals and hypothesis tests were therefore not applied, consistent with the established concern that pseudoreplication can create invalid population-level inference from non-independent observations [21]. No global accuracy, sensitivity, specificity, or false-positive rate was calculated.

#### 3.4.4. Assisted-Operation Output Review and Interlock Chronology

The assisted-operation record extends from 10 July to 4 November 2024. A temporary interlock was used during the controlled industrial test on 23 May and removed at the end of the test. During July, the system underwent controlled corrective observation and configuration management, including work performed during a maintenance lockout. Implementation-specific failure sequences and change details are retained confidentially because they are not necessary to support the scientific claims. The retained records document re-interlocking on 7 August 2024. The configured protective-stop state remained interlocked during the confirmed event on 31 October.

In this study, an output assertion is one protection-related output counted in the consolidated assisted-operation report. It does not necessarily denote an independent physical damage event, a unique conveyor stop, or an independently verified false alarm. The consolidated report assigned fourteen output assertions to six investigation groups. Detailed investigation annexes were used confidentially to verify interlock context, source relationships, and broad operational-maturation categories. Retained historian transitions were corroborative where available and were not treated as a complete independent event ledger. Source differences are disclosed in the Supplementary Materials without reproducing implementation-specific mechanisms, software changes, or corrective algorithms. No false-alarm rate, independent-event count, or occurrence-level reclassification is inferred.

A confirmed real event required concordant optical and control records, an interlocked protective-stop state, field inspection, and maintenance confirmation.

#### 3.4.5. Use of Generative AI Tools

OpenAI ChatGPT (GPT-5.6 Thinking, accessed in July 2026) was used for language and structural assistance. An OpenAI image-generation tool available within ChatGPT (specific model identifier not displayed in the interface, accessed in July 2026) was used for preliminary layouts, icon-based drafts, and visual generation of conceptual figures and the graphical abstract. The physical-arrangement schematic reproduces the previously approved arrangement. The confirmed-event evidence figure was assembled from the retained historian trend, retained event-state record, the original authorized field-inspection photograph, and a separately identified vector reconstruction based on retained event times and reported indications. Generative tools were not used to create or alter its experimental evidence, photograph, numerical data, or event classification. All wording, values, labels, chronology, and final figure compositions were manually checked and edited by the authors against the retained records. The tools did not conduct experiments, collect data, make methodological decisions, independently analyze or interpret results, or formulate the scientific conclusions. The authors take full responsibility for the final content.

## 4. Results

### 4.1. Initial Technical Assessment and Controlled Field Trial

The initial technical assessment documented that the system was not yet mature for protective interlocking. Reported limitations fell into broad environmental, installation, communication, access-control, and configuration-governance categories. These negative findings are retained because they define the starting point for the later commissioning analysis.

During a planned maintenance window on 23 May 2024, five representative artificial defects were produced on the industrial conveyor. With the existing parameterization, the interface visually recognized the manifestations but did not send protective outputs to the PLC during the reported passages. Two defects were then enlarged or added, again without an automatic stop command. After parameter adjustment, the larger anomalies produced protection states. Because the test was qualitative and the per-defect counts were not preserved in a form suitable for statistical analysis, these observations are used only to document the need for redesign and parameter governance.

### 4.2. Belt-Loop Tests

Table 5 presents the controlled repeated-passage results. The 35-mm edge damage remained below the first output threshold and was observed without a PLC-output assertion, consistent with the intended output logic. For the quantified configurations, the observed descriptive proportions depended strongly on geometry and output channel.

The nearly closed central slit was the limiting configuration, with only 39.46% of passages recognized and 20.00% producing a PLC-output assertion. In contrast, the 6-mm V-shaped central opening produced a 96.00% PLC-output proportion. The result supports a geometry-dependent interpretation and does not support pooling all configurations into a single accuracy value. Specifically, the 96.00% entry means that 48 PLC-output assertions were recorded during 50 repeated passages of the same 6-mm V-shaped physical specimen under that test configuration. It does not represent a 96% detection accuracy, sensitivity, or reliability estimate for the monitoring system.

### 4.3. Industrial-Control Integration

The field architecture transferred camera outputs through Modbus RTU, a protocol gateway, and Profibus DP to the plant PLC and supervisory environment. The configured diagnostic and protective outputs were successfully received in the documented integration tests. Communication tests verified diagnostic transport, not detection performance or long-term network reliability.

### 4.4. Assisted Operation, Interlocking, and Corrective Actions

The assisted-operation record covered 10 July to 4 November 2024. Diagnostic activations were dominated by contamination warnings and communication or illumination states. These counts represent state activations, not independent equipment failures. The consolidated report assigned fourteen protection-output assertions to six investigation groups. An assertion does not necessarily denote an independent physical damage event, a unique conveyor stop, or an independently verified false alarm.

The investigation records document operational maturation across broad categories involving communication, environmental robustness, optical-data validity, configuration governance, and internal decision management. The public manuscript intentionally does not reproduce group dates, implementation-specific mechanisms, change sequences, or corrective algorithms because these details are not necessary to audit the reported counts, interlock chronology, or confirmed event. The retained records document controlled corrective observation followed by re-interlocking on 7 August. Detailed source records remain available for confidential editorial review.

Group assignment was not reconstructed from timestamps by the authors. Each assertion was retained under the investigation group assigned in the consolidated assisted-operation report, while detailed investigation records were used only to verify context, source relationships, and evidence boundaries. Where the retained evidence does not establish assertion-level independence, no independent-occurrence classification is inferred. Table 6 provides the public mapping used in the analysis.

### 4.5. Confirmed Industrial Edge-Tear Event

**Figure 4:**
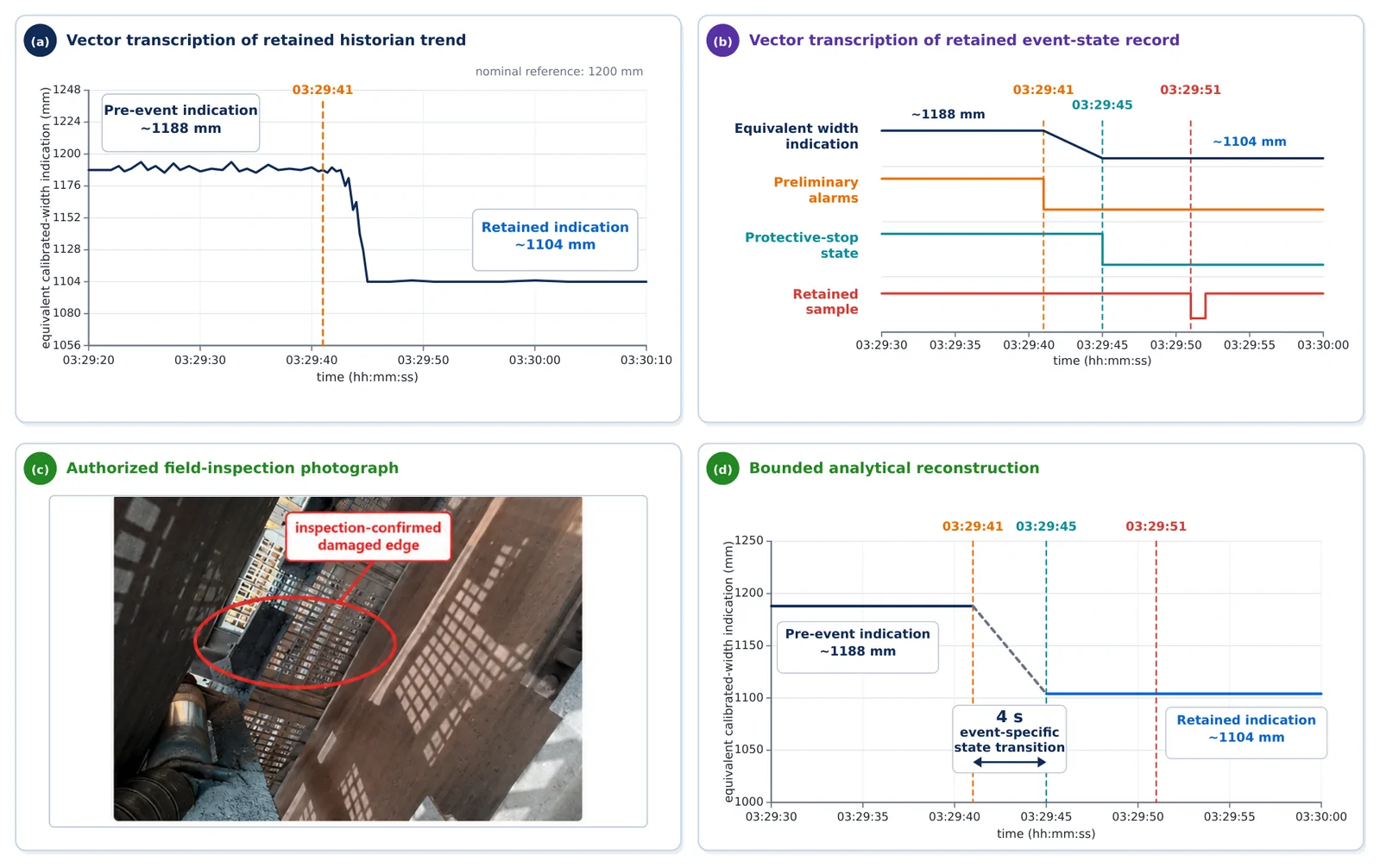
Evidence of the documented 31 October 2024 event. Panels (**a**,**b**) are manually verified vector transcriptions of the authorized retained historian trend and event-state record, respectively, prepared for publication legibility without using the source graphics to derive new measurements. Their event times, state transitions, and equivalent indications are taken from the retained records. Panel (**c**) reproduces the authorized field-inspection photograph from the retained report; the existing red annotation identifies the inspection-confirmed damaged region and was not used for measurement or event classification. Panel (**d**) is a bounded analytical vector reconstruction based only on the retained event times and the two reported indications. The approximate values of 1188 mm and 1104 mm are system indications referenced to the nominal 1200-mm belt width, not independent direct dimensional measurements. The recorded four-second interval covers only the transition from the preliminary alarm states at 03:29:41 to the protective-stop state at 03:29:45. The retained 03:29:51 sample is shown separately. The interval is not an average response-time or full mechanical stopping-time metric.

**Figure 5:**
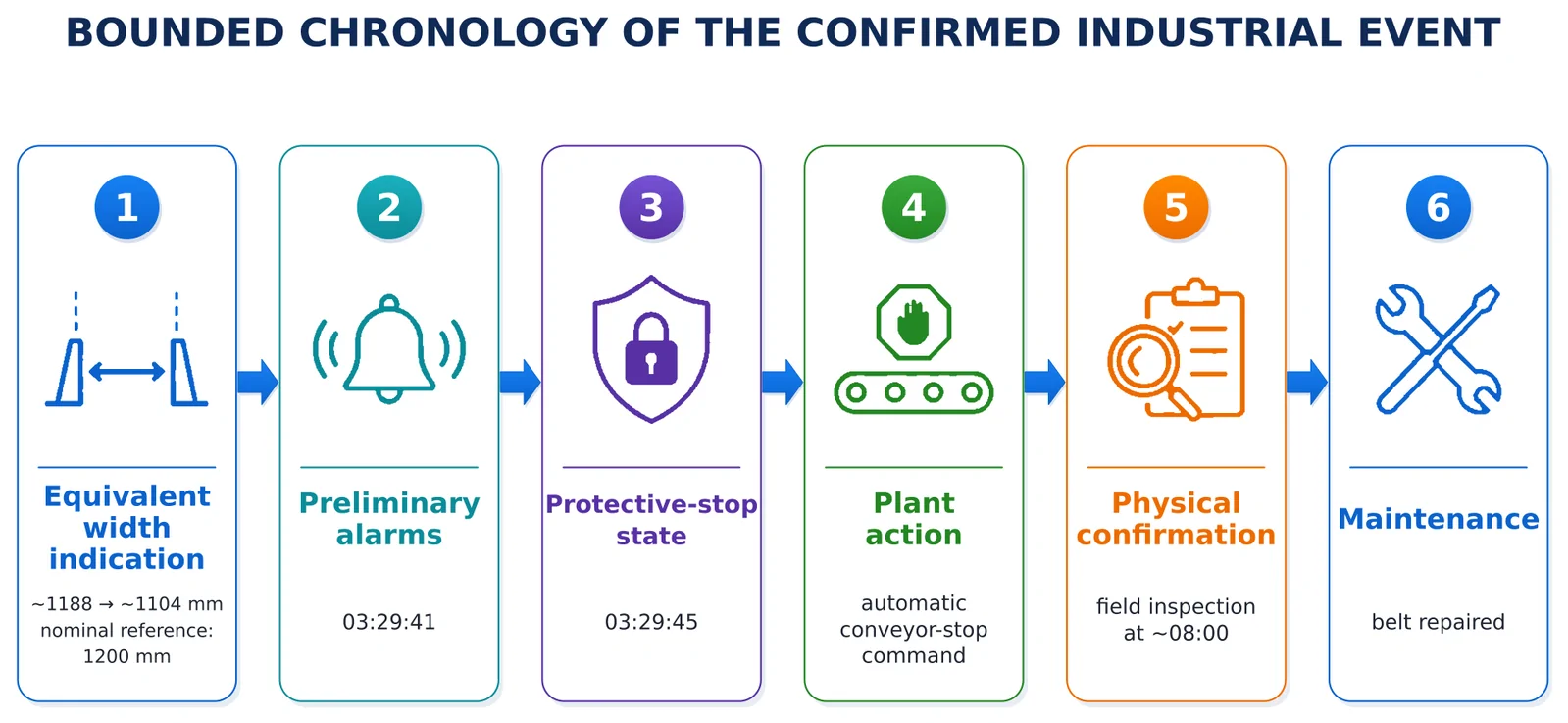
Bounded chronology of the confirmed industrial event. The figure separates the equivalent calibrated-width indication, preliminary alarms, configured protective-stop state, automatic conveyor-stop command, physical confirmation by field inspection, and repair. The approximate values of 1188 mm and 1104 mm are converted system indications referenced to the nominal 1200-mm belt width, not independent direct dimensional measurements.

On 31 October 2024, with the automatic interlock active, the calibrated width indication showed a marked decrease. Using the nominal belt width of 1200 mm as the reporting reference, the retained normalized indications corresponded to approximate calibrated-width equivalents of 1188 mm before the event and 1104 mm in the later retained sample. These converted values are system indications referenced to the commissioned condition and are not independent direct dimensional measurements of the physical belt width. Two preliminary edge-tear alarm states were recorded at 03:29:41. At 03:29:45, the indication reached the configured protective-stop state, which was asserted through the PLC interlock and triggered the automatic conveyor-stop command. Field inspection at approximately 08:00 confirmed an edge tear associated with the monitored manifestation, and maintenance subsequently repaired the belt. The four-second interval belongs only to the transition between the recorded preliminary alarms and the protective-stop state. It is not an average response-time metric and does not represent the full mechanical stopping time of the conveyor. Figure 4 assembles the publication-ready event evidence through vector transcriptions of the retained historian and event-state records, the authorized field-inspection photograph, and a bounded analytical reconstruction. Figure 5 summarizes the documentary event chronology.

A preliminary Portuguese-language conference report presented the open-innovation context of this industrial case at the 4th Latin American Congress of Open Innovation Cases, held in Rio de Janeiro, Brazil, from 13 to 15 August 2025 [22]. The report was published in Volume 3 of the conference proceedings as Case 168 (ISBN 978-65-81033-90-3; doi:10.46848/oiweek2025). The present article substantially extends that report through controlled repeated-passage evidence, source-level reconstruction, industrial-control integration, commissioning analysis, explicit treatment of non-independent observations, and bounded field confirmation. Preliminary performance and economic statements from the conference report are not used as scientific results in this study. No text, figure, table, or artwork from the conference report is reproduced in the present article.

## 5. Discussion

### 5.1. Controlled Repetition Versus Independent Event Performance

Repeated passages provide useful information about within-configuration consistency but do not estimate population-level event sensitivity. Belt markings, illumination, belt motion, and other shared conditions may persist between successive loops and induce dependence among passages. Accordingly, the high proportions observed for the 61-mm edge tear and 6-mm central V opening cannot be generalized to all tears. The low result for the 0.2-mm central slit is equally important because it demonstrates that surface accessibility and opening geometry govern recognition and confirmed-output behavior.

### 5.2. Operational Maturation Through Investigation and Corrective Action

The operational contribution is not an assertion of failure-free or stable performance but the documented progression from investigated outputs to controlled corrective observation and re-interlocking. The retained records show that operational maturity depended on several system layers, including environmental robustness, communication, optical-data validity, configuration governance, and internal decision management. These broad categories are sufficient to distinguish sensing performance from installation, maintenance, communication, and configuration effects. Implementation-specific root causes and corrective algorithms are intentionally not disclosed. Where a record did not document recurrence after a correction, that absence is reported only as a boundary of the retained evidence and is not presented as proof of elimination or as a formal reliability model.

The dirty-camera diagnostic was the most frequent diagnostic condition during the early assisted-operation period, reinforcing evidence that environmental maintenance is central to optical monitoring [10,12]. Likewise, published studies show that sensor orientation and installation geometry can materially affect diagnostic accuracy [11]. The present evidence extends these observations by showing how environmental conditions, image validity, communication, and parameter governance interacted in one industrial deployment.

Two distinct environmental limitations should be separated. First, contamination or optical degradation may cause the internal validity requirements not to be satisfied, rendering the observation unavailable or invalid and producing a diagnostic state rather than a confirmed tear-related output. Second, an observation may satisfy the validity requirements while the accessible manifestation is nevertheless interpreted incorrectly by the decision process. The first is principally a measurement-availability or validity limitation, whereas the second is an interpretation limitation. Their mitigation is correspondingly different: optical maintenance and validity management address the former, while processing and decision robustness address the latter.

### 5.3. Control Transition, Conveyor Inertia, and Protective Interpretation

**Figure 6:**
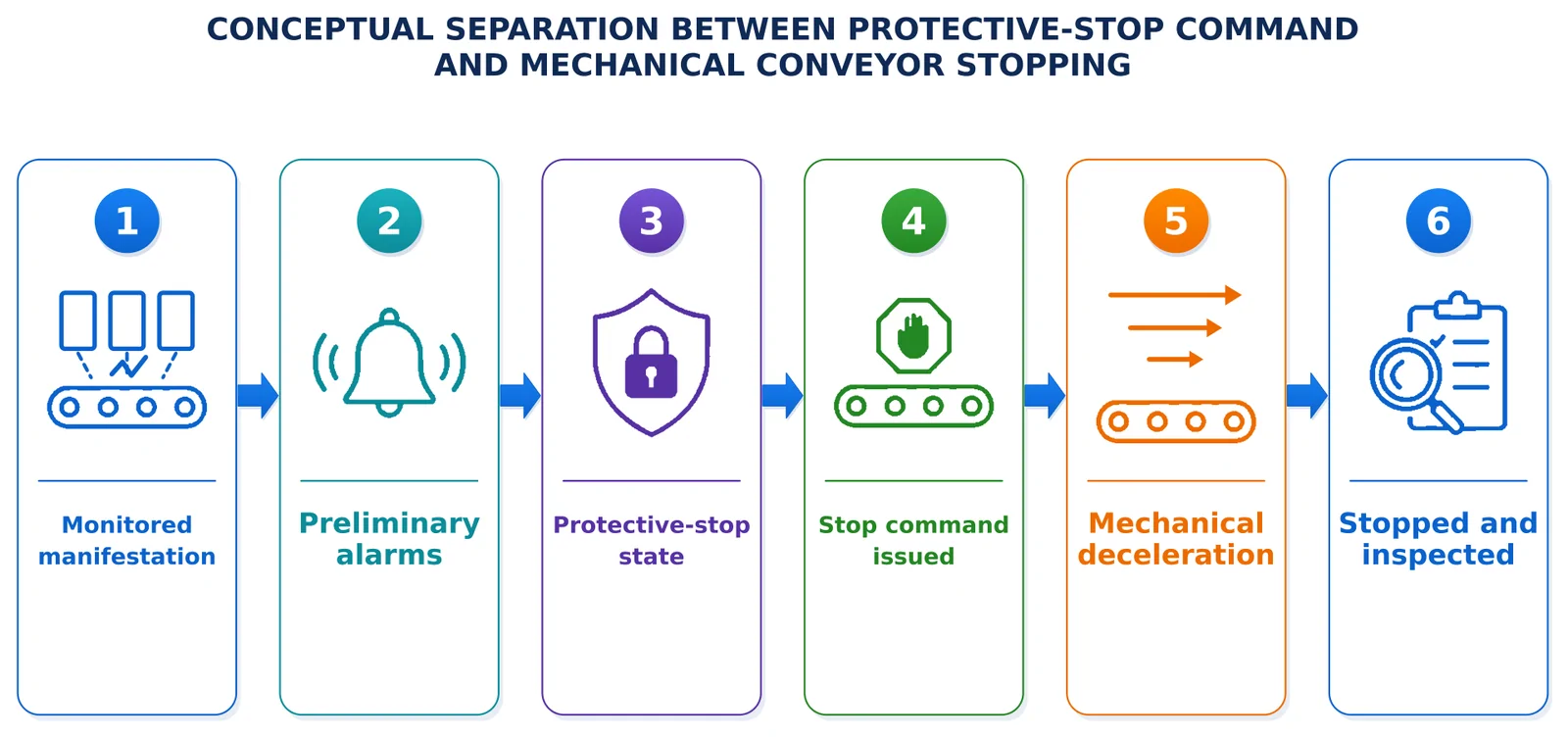
Conceptual separation between the protective-stop command and mechanical conveyor stopping. The six aligned stages separate the monitored manifestation, preliminary alarms, protective-stop state, stop command, mechanical deceleration, and the stopped-and-inspected condition. The documented four-second interval ends at the recorded protective-stop state and is stated in the text rather than repeated inside the figure. Subsequent deceleration depends on drive response, braking, transported load, belt and conveyor properties, and system inertia. The schematic sequence is conceptual and not to scale.

The proprietary internal confirmation logic is one component of the response chain. A complete latency budget would require synchronized timestamps for image acquisition, candidate classification, internal confirmation, communication, PLC processing, drive or brake actuation, and conveyor deceleration. The retained event chronology provides one observed four-second interval between the preliminary alarms and the recorded protective-stop state. This interval marks the recorded sensor-and-control transition leading to the protective command. It does not equal the full time required for the belt to become stationary because mechanical stopping also depends on drive and brake behavior, belt mass, transported load, conveyor length, and system inertia. Figure 6 separates the recorded control transition from subsequent mechanical deceleration. The four-second observation is event-specific and is not a general response-time metric. Similarly, non-actuation of conventional devices in the documented installation cannot establish universal superiority of optical monitoring.

From a protection-engineering perspective, the adequacy of an internal confirmation interval cannot be assessed in isolation. The available protection margin depends jointly on sensor location, belt speed, damage-propagation behavior, communication and control latency, drive or brake response, belt and transported-load inertia, and site-specific stopping criteria. The retained records do not provide a validated allowable braking distance, a tear-propagation law, or synchronized end-to-end stopping measurements suitable for a quantitative protection-margin assessment. Accordingly, no universal or quantitative claim of protective adequacy is made from the internal confirmation process alone.

### 5.4. Controlled-Test Transfer and Morphological Boundaries

The controlled specimens were defined physical challenge geometries intended to provide repeatable observations under the belt-loop conditions; they were not intended to serve as morphological replicas of naturally developing industrial tears. Field damage may include irregular edges, abrasion, material detachment, local deformation, contamination, and evolving opening or propagation under load. Such characteristics can alter optical visibility, accessible geometry, surface texture, and measurement-validity conditions. Consequently, the controlled-test proportions should not be quantitatively extrapolated to the broader population of industrial tear morphologies.

The belt-loop and industrial stages also differed in belt width, belt speed, loading state, environmental contamination, vibration, and surface or optical conditions. These factors may affect both the manifestation available to the optical subsystem and the temporal relationship between passage of a manifestation and the confirmed output. The two stages are therefore treated as complementary rather than dynamically equivalent sources of evidence. The controlled proportions characterize the tested physical specimens and configurations only; no quantitative scale transfer to industrial operation is claimed.

### 5.5. Industrial and Scientific Implications

This single-site case study illustrates a defensible way to organize evidence from a monitoring system that changed during commissioning. Negative tests should be retained because they reveal threshold and configuration limitations. Belt-loop results should be reported by physical scenario. Integration tests should be separated from detection tests. Protection-related occurrences should be linked to dated investigations without retroactively treating every occurrence as an independent false alarm. Finally, a field-confirmed event should remain bounded evidence unless a larger set of events and measured operating exposure are available. The source-traceable structure used here is a case-specific analytical structure, not a validated universal framework. Accordingly, successful transport of diagnostic and protective states through the industrial communication chain is evidence of integration functionality only and is not used as evidence of defect-detection performance. The 31 October occurrence is treated as one documented end-to-end demonstration of the sensing-to-command-to-inspection chain under industrial conditions; it is not interpreted as evidence of general detection reliability.

## 6. Limitations

The controlled passages involved repeated exposure of a small number of physical defect specimens and therefore do not provide independent-event sensitivity. The retained belt-loop report preserved final revolution, recognition, and PLC-counter totals but not synchronized pass-level timestamps, counter-reset logs, ambient measurements, cleaning records between scenarios, or a list of excluded passages. No undocumented exclusions or environmental corrections were therefore introduced. The belt-loop test rig and industrial stages were complementary but not dynamically equivalent. The proprietary confirmation implementation and speed-dependent persistence characteristics were not part of the public analysis; therefore, the study does not establish direct scale transfer or performance for shorter longitudinal manifestations at industrial speed. The central-slit opening was estimated indirectly with a tightly fitting card gauge rather than through a direct calibrated gap measurement, and no formal uncertainty model was retained.

The consolidated assisted-operation report assigned fourteen protection-output assertions to six investigation groups. An assertion is not equivalent to an independent physical event or unique stop, and complete row-level stop records were not retained. Some consolidated group labels differ from timing in the associated detailed annexes. Counts were retained from the consolidated report, while detailed investigations were used confidentially to verify the broad chronology and evidence boundaries without creating unsupported event dates. The study does not estimate a false-alarm subset, false-alarm rate, system availability, or event-by-event stop probability. The public record identifies the functional Modbus RTU–gateway–Profibus DP–PLC chain but intentionally omits the gateway model, register mappings, network configuration, and alternative integration arrangements.

The confirmed industrial event is a single occurrence. Evidence extraction and technical interpretation were performed by authors affiliated with the system supplier and the industrial operator that acquired and deployed the system. No independent institution repeated the tests or validated the classifications. Proprietary design details are omitted to protect intellectual property. The study reports the functional sensing arrangement, monitored regions, externally observable output definitions, functional industrial-integration chain, and control-event sequence. Optical calculation methods, detailed geometry, illumination arrangement, acquisition and confirmation parameters, gateway model, calibration implementation, configuration identifiers, thresholds, register mappings, and source code are not exposed in the publishable record. The study therefore enables audit of the reported input–output evidence and system-level interpretation, but it does not permit implementation-level reconstruction, independent physical replication of the optical subsystem, or component-level replication of the proprietary processing and decision implementation.

The public evidence therefore supports independent verification of the reported counts and derived proportions, review of the functional sensing and industrial-integration architecture, and audit of the stated chronology and claim boundaries using the manuscript, Supplementary Materials, and Data S1. It does not support independent reproduction of the optical measurement implementation, calibration, feature extraction, internal confirmation logic, thresholds, or state-transition implementation. The distinction is therefore between auditability of the reported system-level evidence and reproducibility of the proprietary sensing implementation.

## 7. Conclusions

This retrospective single-site case study reconstructed the staged evaluation of a non-contact optical conveyor-belt monitoring system with on-camera processing. The evidence included an initial technical assessment, negative and positive controlled field results, belt-loop tests, field integration through Modbus RTU, a protocol gateway, and Profibus DP to the plant PLC, assisted-operation investigations, controlled interlock transitions, broad operational-maturation evidence, and one field-confirmed interlocked edge-tear event. The controlled results showed strong dependence on damage geometry: among the quantified configurations, scenario-specific descriptive PLC-output-assertion proportions ranged from 20.0% for an approximately 0.20-mm nearly closed slit to 96.0% for a 6-mm V-shaped opening. These proportions describe repeated passages of specific defects and do not constitute a global accuracy estimate.

The consolidated record supports fourteen protection-output assertions assigned to six investigation groups, without treating them as independent fault events or unique conveyor stops. After controlled corrective observation, the system was re-interlocked on 7 August. On 31 October, the edge-tear indication reached the configured protective-stop state and triggered an automatic conveyor stop, after which inspection confirmed the edge tear and maintenance repaired the belt. No false-alarm subset or rate is reported because the assertions were not independent physical events and the retained records do not provide complete exposure or event-level denominators. The principal methodological lesson is to separate physical manifestation, camera-interface recognition, proprietary internal confirmation, PLC-output assertion, automatic plant action, mechanical stopping, and field confirmation while preserving source discrepancies and evidence limits.

These are case-specific findings from the investigated installation and should not be interpreted as general performance estimates for optical conveyor-belt monitoring systems. The potentially transferable contribution is methodological rather than performance-based: repeated passages of one physical defect should not be treated as independent events, and physical manifestation, sensor recognition, internally confirmed output, PLC assertion, plant action, mechanical stopping, and field confirmation should be maintained as distinct evidence layers. No general reliability, sensitivity, or accuracy claim follows from this single-site dataset. 

## Figures and Tables

**Table 1:** Positioning relative to representative system-level and optical conveyor-belt monitoring studies. Blank or “not reported” entries indicate that the corresponding evidence was not a stated focus of the cited study.

Study	Sensing/Processing	PLC or Industrial Integration	Validation Setting	Negative or Occurrence Analysis	Relevant Distinction
Xu et al. [5]	Binocular line laser and 3D point-cloud processing	Not reported as study focus	Controlled platform	Not reported	Geometric identification and characterization of longitudinal rips
Qiao et al. [6]	Conditional visual features	Real-time system interface	Experimental system	Interference reduction addressed algorithmically	Conditional recognition of tears in the presence of non-tear features
Chamorro et al. [13]	Machine vision plus speed, load, and alignment sensors	PLC conditioning, wireless communication, IoT gateway	Laboratory demonstrator	Communication and operating-condition tests	System-wide health monitoring and multi-protocol integration
Leite et al. [7]	2D laser scanner with statistical change detection	Test-bench data acquisition	Belt test bench	Algorithm comparison	Statistical detection of longitudinal tears from scanner profiles
Wang et al. [14]	Laser scanning, industrial cameras, and YOLOv7	PLC, HMI, remote operation, and dust-control features	Industrially oriented system tests	Environmental robustness considered	Integrated PLC-based surface monitoring for complex environments
Present study	Upper and lower optical camera subsystems with embedded processing and proprietary internal confirmation	Modbus RTU through a protocol gateway to Profibus DP and the plant PLC	Initial assessment, controlled industrial test, belt-loop test rig, assisted operation, and one confirmed event	Six investigation groups covering 14 reported protection-output assertions	Single-site, source-traceable evaluation with negative tests, grouped operational investigations, controlled interlock transitions, source reconciliation, and separation of sensor output from plant action

Abbreviations: HMI, human–machine interface; IoT, Internet of Things; PLC, programmable logic controller.

**Table 2:** Functional measurands and externally observable outputs of the optical subsystems.

Subsystem	Accessible Physical Manifestation	Functional Measurand or State	Externally Observable Output
Face, two upper cameras	Accessible upper-surface edge-position, edge-texture, or local-loss manifestations	Calibrated position, relative-width, lateral-position, texture, and optical-validity indications referenced to the commissioned condition	Valid observation, edge-tear alarm level, protective-level state, misalignment level, or diagnostic
Contraface, one lower camera	Accessible lower-surface central-profile or texture manifestation	Central-profile, texture, and optical-validity indications	Valid observation, central-tear protective-level state, or diagnostic

**Table 3:** Publishable implementation details and explicit replication boundaries.

Element	Documented Public Specification	Boundary
Optical arrangement	Two upper Face cameras and one lower Contraface camera in a fixed industrial installation; calibrated position and surface-texture information support externally observable tear, misalignment, and validity states	Optical geometry, illumination arrangement, camera/lens models, acquisition parameters, standoff, field of view, calibration implementation, and calculation method are not disclosed
Processing and interface	Embedded on-camera processing with diagnostic and protective outputs	Source code, feature extraction, validity rules, thresholds, confirmation parameters, and state-transition logic are proprietary
Internal confirmation	Proprietary confirmation and defect-validation logic was documented in the retained configuration records	Implementation parameters, state logic, and corrective details are not disclosed
Industrial integration	Camera outputs were transferred by Modbus RTU through a protocol gateway to Profibus DP and the plant PLC/supervisory environment	Register addresses, node identifiers, network configuration, and development alternatives are omitted
Configuration traceability	Sequential corrective configurations and controlled re-interlocking were documented	Software version identifiers, build history, parameter values, root-cause details, and implementation-level corrections are retained confidentially

**Table 4:** Staged evidence design.

Stage	Environment	Purpose	Primary Output
Initial technical assessment	Operating mine	Identify physical, logical, communication, configuration-governance, access, and contamination vulnerabilities	Baseline maturity assessment
Controlled field trial	Operating conveyor during planned maintenance	Apply representative artificial defects and test the existing parameterization	Negative and post-adjustment qualitative results
Belt-loop test rig	18.4-m loop, 610-mm belt, 1.9 m/s	Repeated passages of defined edge and central damage geometries	Recognition and PLC-output counts by scenario
Integration	PLC and supervisory environment	Validate the industrial communication interface and PLC/supervisory integration	Successful transfer of configured diagnostic and protective outputs
Assisted operation	Industrial conveyor	Review protection-related occurrences and broad operational-maturation categories	Grouped assertions, interlock chronology, and evidence boundaries
Confirmed event	Industrial conveyor	Reconstruct one real edge-tear occurrence	Optical records, control states, inspection, and repair

**Table 5:** Controlled repeated-passage results. The proportions describe consistency across repeated passages of one physical defect configuration and are not estimates of population-level sensitivity. The two 90-mm rows are output channels evaluated during the same 185 physical passages of the same defect and are not independent tests. The final column is a descriptive count ratio, not a paired conditional probability.

Configuration	$n_{P}$	$n_{R}$	${\hat{p}}_{R}$	$n_{\mathrm{PLC}}$	${\hat{p}}_{\mathrm{PLC}}$	$n_{\mathrm{PLC}}/n_{R}$
Edge tear 61 mm, alarm channel	185	185	100.0%	169	91.35%	91.35%
Edge tear 90 mm, alarm channel	185	185	100.0%	147	79.46%	79.46%
Edge tear 90 mm, protective channel	185	185	100.0%	140	75.68%	75.68%
Central slit approximately 0.20 mm	185	73	39.46%	37	20.00%	50.68%
Central V opening 6 mm	50	50	100.0%	48	96.00%	96.00%

**Table 6:** Public mapping of the fourteen protection-output assertions to the six investigation groups. Group identifiers replace source dates because the public analysis does not reconstruct unsupported event dates.

Group	Assertions	Output Family	Public Independence Interpretation
Group 1	1	Edge tear	One counted assertion. Independent physical-event status is not inferred beyond the retained record.
Group 2	6	Central tear	Six assertions within one investigation group. They are not treated as six independent defect events.
Group 3	2	Edge tear	Two assertions within one investigation group. Assertion-level independence is not established.
Group 4	1	Central tear	One counted assertion. Exact occurrence timing was not independently reconstructed.
Group 5	2	Central tear	Two grouped assertions. Source correspondence remains bounded.
Group 6	2	Central tear	Two grouped assertions. Physical chronology and corrective chronology are not treated as identical.

## Data Availability

Third-party data restrictions apply to the original industrial records, historian exports, images, and internal reports used in this study. These materials are owned by Vale S.A. and LLK Engenharia and are not publicly available because they contain confidential and proprietary information. The data owners have authorized scientific use and confidential editorial review. Original records will be provided to editors and reviewers through the corresponding author upon request using an appropriate secure or confidential channel. Anonymized derived data supporting the reported calculations and claim boundaries are included in the article, Supplementary Materials, and Data S1.

## Supplementary Materials

The following supporting information can be downloaded at: https://www.mdpi.com/article/10.3390/s26185925/s1, Table S1, controlled-test evidence and descriptive calculations; Table S2, commissioning, communication, and interlock chronology; Table S3, anonymized protection-output investigation groups and public evidence boundaries; Table S4, reconciliation of consolidated groups and detailed source records; Table S5, source-level evidence matrix and claim boundaries; Table S6, image-processing and figure-source log; Table S7, derived-claim provenance and verification status; Table S8, public functional specification and disclosure boundary; Table S9, prior-publication and shared-content boundaries; Supplementary Note S1, counting, source reconciliation, interlock, internal-confirmation, and confidentiality boundaries; and Data S1, anonymized derived data and source-boundary workbook. Implementation-specific root causes, corrective algorithms, software versions, internal confirmation parameters, optical calculation methods, and proprietary source records are not included.

## Abbreviations

HMI	Human–machine interface
IoT	Internet of Things
PLC	Programmable logic controller
