# Supplementary material for: Retrospective Staged Evaluation of an Optical Conveyor-Belt Monitoring System with On-Camera Processing: A Single-Site Mining Case Study
Source: Sensors (Basel). 2026 Sep 19;26(18):5925. doi: 10.3390/s26185925 (PMC13611184; doi:10.3390/s26185925)
Supplement: Supplementary file 1 [file sensors-26-05925-s001.zip › Supplementary_Materials.pdf]

# Supplementary Material

## Retrospective Staged Evaluation of an Optical Conveyor-Belt Monitoring System with On-Camera Processing: A Single-Site Mining Case Study

Daniel Luiz Souza et al.

## Scope, source boundaries, and confidentiality

This supplement provides anonymized derived evidence used in the manuscript. Original historian exports, internal reports, source images, proprietary calibration data, optical calculation methods, internal confirmation parameters, decision thresholds, software code, root-cause details, corrective algorithms, and industrial network identifiers are not reproduced. Report labels A–E are analytical identifiers and do not represent public document names. Original records have been authorized for scientific use and confidential editorial review and will be provided to editors or reviewers through a secure channel upon request. The accompanying spreadsheet *Data S1* contains the same derived evidence in machine-readable form. For the confirmed event, the nominal 1200-mm belt width is used only as a reporting reference: retained normalized indications correspond to approximate equivalent calibrated-width indications of 1188 mm and 1104 mm. These converted values are not independent direct dimensional measurements.

## Table S1. Controlled-test evidence and descriptive calculations

| ID | Date / environment | Defect | Dimensions | Passes | Recog. | PLC-output assertions | Interpretation and boundary |
|---|---|---|---|---|---|---|---|
| S1-1 | 14 Jun 2024 / belt-loop test rig | Edge tear below alarm threshold | 35 × 1000 mm, nominal | NR | Observed | 0 | Intended suppression below the first output threshold. Number of passages was not preserved. |
| S1-2 | 14 Jun 2024 / belt-loop test rig | Edge tear, alarm threshold | 61 × 1000 mm, nominal | 185 | 185 | 169 | Recognition 100.0%; PLC-output assertion 91.35%. Repeated passages of one physical defect. |
| S1-3 | 14 Jun 2024 / belt-loop test rig | Edge tear, alarm channel | 90 × 1000 mm, nominal | 185 | 185 | 147 | Recognition 100.0%; PLC-output assertion 79.46%. Same physical passages as S1-4. |
| S1-4 | 14 Jun 2024 / belt-loop test rig | Edge tear, protective channel | 90 × 1000 mm, nominal | 185 | 185 | 140 | Recognition 100.0%; PLC-output assertion 75.68%. Same physical passages as S1-3. |
| S1-5 | 14 Jun 2024 / belt-loop test rig | Nearly closed central slit | Approximately 0.20 × 1000 mm | 185 | 73 | 37 | Recognition 39.46%; PLC-output assertion 20.00%; PLC-output-to-recognition count ratio 50.68%. Opening estimated from a tightly fitting card whose thickness was $0.20 \pm 0.02$ mm over five caliper measurements; the slit itself was not directly measured. |
| S1-6 | 23 Jun 2024 / belt-loop test rig | Central V opening | 6 × 1000 mm, nominal | 50 | 50 | 48 | Recognition 100.0%; PLC-output assertion 96.00%. Repeated passages of one physical defect. |

*Abbreviation: PLC, programmable logic controller.*

**Calculation rule.** For scenario $j$, the descriptive recognition and PLC-output proportions are

$$\hat{p}_{R,j} = n_{R,j}/n_{P,j}, \qquad \hat{p}_{PLC,j} = n_{PLC,j}/n_{P,j}.$$

No confidence interval or population-level sensitivity estimate is reported because repeated passages of the same specimen are non-independent repeated observations rather than independent experimental replicates. The recognition counts were taken from documented camera-system interface counters and the confirmed-output counts from dedicated PLC counters. The retained report preserves final scenario totals but not synchronized pass-level timestamps, counter-reset logs, ambient measurements, cleaning records between scenarios, or discarded-passage records;

no undocumented exclusions or environmental corrections were introduced. The approximately 0.20-mm central opening was not measured directly. A rigid card was inserted with a tight fit, and five caliper measurements of its thickness yielded $0.20 \pm 0.02$ mm. This dispersion characterizes the card measurements and is not interpreted as a formal uncertainty of the slit opening. The alarm-channel and protective-channel rows for the 90-mm edge defect represent two output channels evaluated during the same 185 physical passages.

# Table S2. Commissioning, communication, and interlock chronology

| Period / date | System or operating state | Automatic interlock status | Evidence retained | Interpretive boundary |
|---|---|---|---|---|
| Through Mar 2024 | Initial technical assessment and development | System judged not mature for permanent protective interlocking | Initial technical assessment | Baseline only; no post-correction performance claim |
| 23 May 2024 | Controlled industrial test during planned maintenance | Temporarily connected for the test; protections removed at test completion | Controlled field-trial record | Temporary trial does not establish continuous field use |
| 26 Jun–16 Jul 2024 | Definitive control-interface implementation and integration | Integration developed before renewed interlocking | Integration record | Communication testing is not detection performance |
| July 2024 | Protection-output investigations and controlled corrective observation | Interlock status changed under documented control | Consolidated assisted-operation report and detailed records | Counts are public; implementation-specific mechanisms remain confidential |
| July 2024 | Configuration work during a maintenance lockout and subsequent corrective observation | Managed under the documented commissioning process | Detailed confidential investigation and consolidated report | No implementation-specific change sequence or corrective implementation is reproduced publicly |
| July 2024 | Corrective configuration observed before renewed plant protection | Withdrawn during corrective observation | Detailed communication and observation record | No inference about continuous device exposure |
| 7 Aug 2024 | Corrected configuration returned to plant protection | Re-enabled | Detailed corrective-action record | Exact intra-day activation time not retained |
| 31 Oct 2024 | Confirmed edge tear reached the configured protective-stop state, initiated the automatic conveyor-stop command, was inspected, and was repaired | Active | Optical/control record, field inspection, and maintenance confirmation | One confirmed event; no population-level performance inference |
| Field communication | Modbus RTU through a protocol gateway to Profibus DP and the plant PLC/supervisory environment | Applicable to documented field installation | Integration and assisted-operation records | Register mappings, network identifiers, and alternative integration arrangements retained confidentially |

*Abbreviation: PLC, programmable logic controller.*

# Table S3. Anonymized protection-output investigation groups and public evidence boundaries

| Group | No. | Output family | Public analytical treatment | Confidential boundary |
|---|---|---|---|---|
| Group 1 | 1 | Edge tear | One protection-output assertion retained in the consolidated count | Root-cause detail, configuration state, and corrective implementation omitted |
| Group 2 | 6 | Central tear | Six assertions assigned to one investigation group, not six independent defect specimens | Update sequence, configuration mechanism, and corrective procedure omitted |
| Group 3 | 2 | Edge tear | Two assertions retained within one investigated group | Environmental and processing implementation details omitted |
| Group 4 | 1 | Central tear | One assertion retained; exact occurrence date was not reconstructed | Detailed processing mechanism and corrective algorithm omitted |
| Group 5 | 2 | Central tear | Two assertions retained; source correspondence remains bounded | Optical-data-validity mechanism and corrective implementation omitted |
| Group 6 | 2 | Central tear | Two assertions retained; physical chronology and corrective date are not treated as identical | Internal decision-management mechanism and corrective implementation omitted |

An *output assertion* is one protection-related output counted in the consolidated assisted-operation report. It does not necessarily denote an independent physical damage event, a unique conveyor stop, or an independently verified false alarm. Counts are retained from the consolidated report. Detailed investigation records were used confidentially to verify chronology and evidence boundaries; implementation-specific mechanisms and corrective actions are not reproduced in the public supplement.

# Table S4. Reconciliation of consolidated groups and detailed source records

| Group | Consolidated count | Source relationship | Analytical treatment | Excluded inference |
|---|---|---|---|---|
| Group 1 | 1 | Consolidated and detailed sources aligned at the investigation level | Count and interlock context retained | No generalized stop probability |
| Group 2 | 6 | Consolidated and detailed sources aligned as one investigation group | Included within the total of fourteen | Not six independent defects and not an additional incident |
| Group 3 | 2 | Consolidated and detailed sources aligned at group level | Count retained | No independent-event sensitivity |
| Group 4 | 1 | Detailed source did not preserve a defensible occurrence date | Consolidated grouping retained | Report date not converted into event date |
| Group 5 | 2 | Detailed source relationship to the consolidated group remains unresolved | Count retained with explicit source limitation | No silent harmonization or direct event linkage |
| Group 6 | 2 | Detection, inspection, and corrective records use different dates | Physical sequence bounded without assigning one unsupported date | Corrective date not represented as detection date |

## Table S5. Source-level evidence matrix and claim boundaries

| Source | Period / setting | Evidence used | Claim supported | Claim not supported |
|---|---|---|---|---|
| Report A | Through Mar 2024 / operating mine | Initial physical, logical, communication, contamination, configuration-governance, and access-control vulnerabilities | Baseline technical assessment | Quantitative post-correction performance |
| Report B | 23 May 2024 / controlled industrial test | Temporary interlock, artificial defects, negative result with existing parameters, and qualitative output after adjustment | Need for redesign and parameter governance | Per-defect sensitivity or continuous interlocked operation |
| Report C | Jun–Sep 2024 / PLC and network integration | Modbus RTU–gateway–Profibus DP communication and PLC/supervisory integration | Communication functionality and transfer of configured diagnostic and protective outputs | Register mappings, network configuration, and alternative integration arrangements |
| Report D | 14 and 23 Jun 2024 / belt-loop test rig | Defined damage geometries, repeated passages, recognition, and PLC-output counts | Scenario-specific repeated-pass consistency | Independent-event sensitivity, specificity, global accuracy, or internal confirmation implementation |
| Report E | 10 Jul–4 Nov 2024 / assisted operation | Consolidated group counts, confidential investigation verification, interlock chronology, confirmed event, inspection, and repair | Bounded commissioning chronology and one field-confirmed event | Availability, stationary false-alarm rate, complete event ledger, or average response time |
| Data S1 | Derived workbook | Recomputed proportions, chronology, date reconciliation, and claim audit | Publicly auditable derived calculations and boundaries | Original confidential records |

# Table S6. Image-processing and figure-source log

| Figure | Source type | Processing applied for publication | Content boundary |
|---|---|---|---|
| Main Figure 1 | Conceptual vector layout prepared with generative-image assistance and manually redrawn and verified | Vector reconstruction and manual correction of labels, hierarchy, spacing, icon geometry, and scientific wording | Explanatory framework; not a raw measurement |
| Main Figure 2 | Simplified functional physical-arrangement diagram | Camera count, longitudinal station, monitored surfaces, and subsystem labels retained | Optical geometry, illumination arrangement, field of view, acquisition parameters, and internal processing omitted |
| Main Figure 3 | Functional architecture diagram prepared with generative-image assistance and manually verified | Protocol-level communication chain retained; protocol gateway and protocol-conversion function shown; internal confirmation wording generalized | Gateway model, register maps, network identifiers, thresholds, and implementation omitted |
| Main Figure 4a and 4b | Manually verified vector transcriptions of the retained historian trend and retained event-state record | Recorded curves, states, and equivalent indications retained in publication-legible vector form; preliminary alarms at 03:29:41, protective-stop state at 03:29:45, and the separate 03:29:51 retained sample preserved; no new measurement derived from the source graphics | Proprietary calibrated-width implementation omitted; no alteration of the retained trend or state chronology |
| Main Figure 4d | Bounded vector reconstruction from retained event times and reported indications | Four-second alarm-to-protective-state transition and later retained indication shown separately | Explicitly identified as reconstruction; not a raw screenshot, average response-time metric, or full stopping-time reconstruction |
| Main Figure 4c | Authorized field-inspection photograph | Crop and existing explanatory annotation retained | Annotation identifies the inspection-confirmed region only and was not used for measurement or classification |
| Main Figure 5 | Bounded event chronology vector prepared with generative-image assistance and manually redrawn and verified | Percentage values removed; externally observable sequence retained in the approved vector layout | No proprietary width-calculation or threshold information |
| Main Figure 6 | Conceptual stopping-chain vector prepared with generative-image assistance and manually redrawn and verified | Separates recorded control transition from mechanical deceleration and final stop in the approved vector layout | Explanatory, not a measured stopping-time reconstruction |
| Graphical abstract | Five-stage graphical summary prepared with generative-image assistance and manually verified against the final Version 17 text and figures | Final protocol chain, Day 1/Day 106 operational span, 14 assertions in 6 investigations, automatic stop, and field confirmation retained | No specific dates, internal confirmation parameters, calibrated-width values, new result, measurement, or implementation detail |

# Table S7. Derived-claim provenance and verification status

| Derived claim | Primary source type | Verification | Boundary retained in manuscript |
|---|---|---|---|
| Controlled passage proportions | Belt-loop record and PLC counter tables | Integer counts independently recalculated | Descriptive proportions only; no independent-event inference |
| Six groups / fourteen assertions | Consolidated assisted-operation report | Counts summed to fourteen and reconciled with six labeled groups | Assertions are not independent fault specimens, unique stops, or a false-alarm rate |
| Corrective-observation chronology | Detailed confidential investigations and consolidated group record | Group counts and broad interlock chronology cross-checked | No public disclosure of implementation-specific change sequence or root cause |
| Interlock chronology | Controlled trial and detailed July/August annexes | Temporary 23 May connection, controlled July observation, and re-interlock on 7 Aug cross-checked | No claim of uninterrupted interlocked operation |
| Field communication path | Integration and final field records | Industrial communication and PLC integration reconciled | Register mappings, network identifiers, and alternatives retained confidentially |
| 31 Oct confirmed event | Optical/control records, inspection, and repair | Recorded times, calibrated-width indication change, protective-stop interlock, automatic stop, inspection, and repair cross-checked | One event; four seconds is not an average response time |
| Central-slit opening | Controlled-test record | Tight card-gauge procedure checked | Approximately 0.20 mm; no direct gap measurement or formal uncertainty interval |
| Investigation-detail boundary | Detailed confidential investigations | Broad group chronology and count verified without publishing implementation-specific mechanisms | No root-cause, corrective-algorithm, or software-history disclosure |
| Public system specification | Reports A, C, D, and detailed corrective annexes | Publish only the functional architecture and auditable input–output evidence | No component-level, algorithmic, configuration, or source-code reproducibility claim |
| Oiweek 2025 claims | Preliminary conference case and primary records | Exclude unverified economic, preservation, generalized accuracy, predictive-maintenance, and response-time statements | Retained as a cited preliminary communication; claims excluded from scientific results |

# Table S8. Public functional specification and disclosure boundary

| Element / version | Documented information | Function or corrective scope | Public boundary |
|---|---|---|---|
| Optical station | Two upper Face cameras and one lower Contraface camera in a fixed industrial installation | Upper- and lower-surface monitoring | Optical geometry, illumination arrangement, acquisition parameters, calibration, and internal image processing are not disclosed |
| Embedded processing | Proprietary recognition, optical-data-validity management, and internal confirmation | Diagnostic and protective output generation | Source code, feature extraction, thresholds, sample count, timing implementation, persistence characteristics, and state logic are proprietary |
| Industrial integration | Modbus RTU through a protocol gateway to Profibus DP and the plant PLC/supervisory environment | Transfer of configured diagnostic and protective outputs | Register assignments, network identifiers, and alternative arrangements are omitted |
| Configuration management | Sequential corrective configurations and controlled re-interlocking were documented | Operational maturation and source traceability | Version identifiers, parameter values, build history, and implementation-level corrections are retained confidentially |
| Protective-stop state | System-specific protective output documented in the retained installation records | Configured to initiate the automatic conveyor-stop function | Internal nomenclature and intensity thresholds are not disclosed |

# Table S9. Prior-publication and shared-content boundaries

| Related output | Shared factual basis | Content not imported as evidence in the present study | Editorial treatment |
|---|---|---|---|
| Oiweek 2025 conference case | Same open-innovation deployment and simplified description of automatic stopping | 70% belt-preservation estimate, savings up to 95%, detection within three seconds, generalized high-accuracy language, predictive-maintenance characterization, and replication claims | Cited as preliminary communication; claims excluded from scientific results and claim audit |

## Supplementary Note S1. Counting, source reconciliation, interlock, internal-confirmation, and confidentiality boundaries

1. The controlled-test passage count $n_P$ was the final documented revolution count: 185 revolutions for each quantified 14 June scenario and 50 revolutions during 8 min 10 s for the 23 June V-opening scenario. One passage represented one completed revolution of the same physical defect through the fixed sensing station. The recognition count $n_R$ was taken from documented camera-system interface counters, and $n_{PLC}$ was taken from dedicated PLC counters. The retained report does not preserve synchronized pass timestamps, counter-reset logs, pass-level operator decisions, ambient measurements, cleaning records between scenarios, or discarded-passage records; no undocumented exclusions were introduced.
2. Repeated passages of the same physical defect are non-independent repeated descriptive observations and are not independent experimental replicates.
3. An output assertion is one protection-related output counted in the consolidated assisted-operation report. It is not automatically an independent physical event, a unique conveyor stop, or an independently verified false alarm.
4. Counts and the six anonymized group identifiers are taken from the consolidated assisted-operation report. Detailed annexes were used confidentially to verify chronology and source boundaries. Source discrepancies are disclosed without reproducing implementation-specific mechanisms or corrective actions.
5. The six assertions in Group 2 belong to one investigation group and are included in the reported total of fourteen. They are not treated as six independent defects or as an additional incident outside the consolidated chronology.
6. The retained sources support temporary interlocking on 23 May, controlled July interlock transitions, re-interlocking on 7 August, and active automatic interlocking on 31 October. They do not support a claim of uninterrupted interlocked operation across the entire period.
7. The field installation transferred camera outputs through Modbus RTU, a protocol gateway, and Profibus DP to the plant PLC and supervisory environment. The register mappings, network identifiers, and alternative integration arrangements are retained confidentially.
8. The retained records document embedded processing and proprietary internal confirmation and defect-validation logic. Implementation parameters, state logic, calculation details, and corrective implementation are not disclosed.
9. Bench and industrial stages are complementary rather than dynamically equivalent. Direct scale transfer and implementation-level confirmation characteristics are outside the public evidence boundary.
10. A confirmed real event required optical and control records, an interlocked stop state, field inspection, and maintenance confirmation.
11. No global accuracy, independent-event sensitivity, specificity, false-alarm rate, availability, event-by-event stop probability, or stationary reliability estimate is inferred.
12. The public specification supports audit of the input–output evidence and system-level interpretation of the sensing/control chain, but camera/lens models, pixel resolution, laser wavelength, exact geometry, calibration constants, thresholds, and source code are not exposed; independent physical replication of the optical subsystem and component-level replication of the proprietary processing are not claimed.
13. The Oiweek 2025 performance and economic statements listed in Table S9 are not used as evidence in the present study.
